# An expanded polyglutamine in ATAXIN1 results in a loss-of-function that exacerbates severity of Multiple Sclerosis in an EAE mouse model

**DOI:** 10.21203/rs.3.rs-5664390/v1

**Published:** 2025-04-14

**Authors:** Gourango Talukdar, Lisa Duvick, Praseuth Yang, Brennon O’Callaghan, Gavin J. Fuchs, Marija Cvetanovic, Harry T. Orr

**Affiliations:** University of Minnesota; University of Minnesota; University of Minnesota; University of Minnesota; University of Minnesota; University of Minnesota; University of Minnesota

**Keywords:** ATAXIN1, Multiple sclerosis, autoimmune, demyelination, EAE, SCA1

## Abstract

**Background and Objectives:**

Ataxin-1 (ATXN1) is a protein in which expansion of its polyglutamine tract causes the neurodegenerative disorder spinocerebellar ataxia type 1 (SCA1) via a gain-of-function. Wild type ATXN1 was recently shown to have a protective role in regulating severity of experimental autoimmune encephalomyelitis (EAE), a well-established mouse model for Multiple sclerosis (MS). This study further investigates the role of ATXN1 with an expanded polyglutamine tract in the context of MS using an EAE mouse model.

**Methods:**

Hemizygous *Atxn1 (Atxn1*^*2Q/*−^) mice or *f*-*ATXN1*^*146Q/2Q*^, heterozygous mice that have one copy of the endogenous mouse gene replaced with a polyQ expanded pathogenic human *ATXN1* gene, were injected with myelin oligodendrocytes glycoprotein (MOG_35 – 55_) peptide to induce EAE. Immunohistochemical and biochemical approaches were used to analyze the degree of demyelination, cell loss, axonal degeneration as well as detecting the activated immune cells and inflammatory cytokines upon EAE induction in *Atxn1*^*2Q/*−^ and *f*-*ATXN1*^*146Q/2Q*^ mice.

**Results:**

Our findings reveal that a loss-of-function of wild type *Atxn1* in *Atxn1*^*2Q/*−^ and *f-ATXN1*^*146Q/2Q*^ mice significantly exacerbates the EAE symptoms, leading to increased demyelination, oligodendrocytes loss, heightened axon degeneration, and greater clinical disability in affected mice. Importantly, the data reveals that neurotoxic astrocytes are activated at acute stage of disease (PID-14) and at the chronic stage of disease (PID-30) neurotoxic astrocytes no longer show signs of activation. The data also demonstrated enhanced infiltration of immune cells into the lesions of mutant mice.

**Discussion:**

These results indicate that ATXN1 plays a protective role in modulating immune responses and maintaining neural integrity during MS. Importantly, expansion of the polyQ tract in ATXN1 results in a loss-of-function in ATXN1’s ability to dampen the immune response. Understanding the functional role of ATXN1 in MS pathogenesis may open new avenues for therapeutic strategies aimed at mitigating disease progression.

## Introduction

Expansion of the polyglutamine tract in ATAXIN1 (ATXN1), a nuclear protein^1^, causes spinocerebellar ataxia type 1 (SCA1), a heritable neurodegenerative disorder^2,3^. Neuroinflammation is a common feature of neurodegenerative disorders^4–6^. Recently, the *ATXN1* gene was identified as a susceptibility locus for multiple sclerosis (MS)^7–10^, a multifaceted autoimmune disorder characterized by chronic inflammation, demyelination, and subsequent neuronal damage within the central nervous system (CNS)^11–13^. MS presents a wide spectrum of symptoms, ranging from focal inflammation to neuronal death, axonal and myelin loss, and failure of CNS repair mechanisms to restore the damage^14^. MRI studies show that cortical demyelination is common in early-stage MS, with approximately 30% of patients with a clinically isolated syndrome exhibiting cortical lesions^15^. Furthermore, MS is traditionally viewed as a chronic inflammatory disease of the CNS, leading to the formation of focal demyelinated plaques in white matter^16^. Pathology of MS emphasizes the demyelinating aspects of the disease process, with a preservation of axons in the lesion area^17^. In MS, CNS pathology extends beyond white matter, with grey matter damage occurring early in the disease evolution, correlating with clinical disability and cognitive dysfunction^18^.

Regulation of B cell function, B cell receptor signaling, and the expression of specific noncoding RNAs in B cells upon autoimmune demyelination were shown to be aspects of ATXN1’s involvement in the pathogenesis of MS^7–10^. The identification of *ATXN1* as a susceptibility gene for MS underscores the importance of further understanding its role in the disease process and its potential as a therapeutic target. However, the function of ATXN1 in the pathogenesis and progression of MS in the CNS remains elusive. In the present study, we investigate the role of ATXN1 in CNS autoimmunity, specifically in the pathophysiology of MS diseases progression. We employ the experimental autoimmune encephalomyelitis (EAE) mouse model, to further explore the role of ATXN1 in MS pathogenesis. Our findings indicate that the loss-of-function (via heterozygous knockout or 146Q expansion) of ATXN1 increases autoimmune demyelination, axon degeneration, and oligodendrocyte loss which, is associated with the activation of immune cells and inflammatory cytokines in the site of CNS lesion.

## Materials and Methods

### Mice

*f-ATXN1*^*146Q/2Q*^ mice are a conditional knock-in mouse model where the coding exons of one allele of the mouse *Atxn1* gene was replaced with the human *ATXN1* coding exons using site-specific recombination at flanking FRT and LoxN recombination sites^19^. *Atxn1*^*2Q/*−^ mice, heterozygous SCA1 null mice, were generated as described^20^. The University of Minnesota Institutional Animal Care and Use Committee approved all animal use protocols. Mice were housed and managed by Research Animal Resources under specific pathogen-free conditions in an Association for Assessment and Accreditation of Laboratory Animal Care International approved facility. Food and water were provided *ad libitum*. All mice were age matched (8–9 weeks) within experiments and littermate controls (*Atxn1*^*2Q/2Q*^) were used.

Female mice were used for EAE experiments because the MOG-induced EAE model is well established to exhibit a stronger and more consistent disease phenotype in females, reflecting the higher prevalence of MS in women^21^ and male mice were used for characterization. All mice were maintained on a C57BL/6 genetic background. Samples were collected at post-immunization day 14 (PID-14, acute phase) and 30 (PID-30, chronic phase) of the disease, representing the relapse and remission states, respectively. We chose PID-14 as it marks the initiation of peak disease progression, which occurs at PID-16/17.

### EAE immunization

Eight-week-old female mice were anesthetized using 1.8% isoflurane and injected subcutaneously in the flank/tail base with 200mg of MOG_35 – 55_ peptide (Genemed Synthesis Inc.) emulsified in complete Freund’s adjuvant (BD Biosciences) supplemented with 600mg of *Mycobacterium tuberculosis* (strain H37Ra; BD Biosciences). Two intraperitoneal injections of 400ng pertussis toxin (Biological Laboratories) were given 0 and 48h later. Clinical scores (0 = healthy, 0.5 = tail shows slight limpness, 1 = flaccid tail, 1.5 = weak hind limbs, 2 = ataxia paresis of hind limbs with abnormal gait, 2.2 = wedge gait duck walk, 2.3 = leg paresis/drag one leg, 2.5 = paralysis of one leg, 2.8 = drag one leg, paralysis another leg, 3 = paralysis of hindlimbs and/or paresis of forelimbs, 3.5 = paralysis of hind limbs, paralysis of one front limb, 4 = tetra-paralysis, 5 = moribund or death) were recorded daily as described previously^21,22^. The aggregate EAE clinical score was the sum of daily clinical scores for each individual mouse during the observation period.

### Immunohistochemistry (IHC)

Mice were deeply anesthetized with ketamine and xylazine cocktail by intraperitoneal injection and perfused through the left cardiac ventricle with PBS (in 0.4 mg/ml heparin) followed by 10% buffered formalin phosphate. Half sagittal brain and the cephalic half of the lumbar spinal cord (SC; L1–L3) were postfixed in 10% buffered formalin phosphate for 2h, cryoprotected in 30% sucrose for 48h, embedded in optimum cutting temperature compound, and frozen on dry ice. Frozen sections were cut using a cryostat at a thickness of 16μm. The other half sagittal brain and the caudal half of the lumbar SC (L3–L5) were postfixed in 10% buffered formalin phosphate for 72h, dehydrated through graded alcohols, and embedded in paraffin wax. Paraffin sections were cut using a microtome to a thickness of 5μm. For immunofluorescence, the frozen sections were treated with − 20°C acetone and paraffin section were deparaffinized by treating with xylene and hydrated. The samples were then blocked with PBS containing 10% goat/horse serum and 0.1% Triton X-100, and incubated overnight with the primary antibody diluted in blocking solution.

We used the following primary and secondary antibodies for immunohistochemical detection: CC1 (APC7, 1:50; Millipore, RRID:AB_2057371), myelin basic protein (MBP,1:1000; BioLegend, RRID:AB_2616694), CD3 (1:50; BioLegend, RRID:AB_312658), NeuN (1:500; Abcam, RRID:AB_10711040), glial fibrillary acidic protein (GFAP; 1:200; Agilent Technologies, RRID:AB_10013382), Iba1 (1:200; FUJIFILM Wako’s, RRID:AB_839504), and Neurofilament H (NF-H) (1:200; BioLegend, RRID:AB_32715852). Fluorescein (1:200, Thermo Fisher Scientific, anti-rabbit, RRID:AB_2534088; anti-mouse, RRID:AB_2576217), Cy3 (1:200, Jackson ImmunoResearch Labs, anti-rabbit, RRID:AB_2338006), or enzyme-labeled secondary antibodies (1:200, Vector Laboratories, anti-mouse/rabbit, RRID:AB_2336826). Finally, sections were mounted in ProLong Gold Antifade with 4′,6- diamidino-2-phenylindole (DAPI) to visualize nuclei (Thermo Fisher Scientific) for immunofluorescence and in toluene for 3,3’-Diaminobenzidine (DAB) (Vactor laboratories) staining. Immunofluorescence images were acquired on a Leica Stellaris 8 microscope equipped with a Leica HC PLAN APO 63X objective and stitched together with LASX software (Leica) and DAB images were acquired on a Zeiss Axioskop II to allow visualization of the lumbar SC. To quantify cells and axons in the white matter of lumbar spinal cord, we counted immune-positive cells or axons in an area of 0.1mm^2^ within the anterior funiculus medially next to the anterior median fissure in the lumbar SC as described previously^21,22^. Demyelination and number of cells were quantified using Fiji software. To analyze the cell body of Iba1^+^ cells we counted both hypertrophic and amoeboid cells using Halo software.

### RT-qPCR

The lumbar spinal cord from each mouse was homogenized in 500μL TRIzol Reagent (Thermo Fisher Scientific, 15596026). RNA isolation was done per the manufacturer’s instructions^19^. cDNA was synthesized in duplicate using 500ng RNA in 10μL iScript Advanced cDNA Synthesis Kit (Bio-Rad, 172–5038). Reactions were diluted 1:5 with water. RT-qPCR was done using 2μL diluted cDNA in 10μL Roche Probes Master (04707494001) reactions on a Roche 480 Lightcycler. Target gene and reference gene reactions were amplified in separate wells under cycling conditions of 95°C for 10s, 60°C for 10s for 35 cycles. Cq (quantitation cycle) values were determined using the Roche second derivative maximum calculation. Relative quantification was done using standard 2^ΔΔCq19^.

Primers used include GFAP forward (5’-AGTTGCAGTCCTTGACCTG-3’) and GFAP reverse (5’-CAGCGCCTCCTGATAACTG-3’); C3 forward (5’-CCTTCCACCTTTTTCCTTCACT-3’) and C3 reverse (5’-CTCCAGCCGTAGGACATTG-3’); Clcf1 forward (5’-CCATCCAGAAAACCTATGACCT-3’) and Clcf1 reverse (5’-GATTGAAGTCAGGCTCGTTGA-3’); Slc1a2 forward (5’-CCATGCTCCTCATTCTCACAG-3’) and Slc1a2 reverse (5’-AAAGAATCGCCCACCACAT-3’); TNFα forward (5’-TTGGTCTGATTGTTGGAGTGA-3’) and TNFα reverse (5’-CTTGGCATCTCTTTGTTAGGCA-3’) with probe (5’-/56-FAM/TGCTGATGT/ZEN/TAGGACTGGTGAACTGC/3IABkFQ/-3’); INFγ forward (5’-AGTAGTTATCCTGGTATTTGCGT-3’) and reverse (5’-TTGTCTCTAACGTGGCACTT-3’) with probe (5’-/56-FAM/AATGTTACC/ZEN/TAAGTCCTTGCTCTCTGTGG /3IABkFQ/-3’); IL-17 forward (5’-GCTGCCTAAATGACTGTTTGAG-3’) and IL-17 reverse (5’-AGAATGGCGATGAGTGTGATG-3’) with probe (5’-/56-FAM/ CTGGCTTGG/ZEN/GAACTGTGGTATTTGAGA/3IABkFQ/-3’); iNOS forward (5’-GATCCAGTGGTCCAACCTG-3’) and iNOS reverse (5’-GACCTGATGTTGCCATTGTTG-3’) with probe (5’-/56-FAM/CAGATGTGC/ZEN /TGAAACATTTCCTGTGCT/3IABkFQ/-3’); IL-10 forward (5’-CGGAGACTACACTGTGAGAGT-3’) and IL-10 reverse (5’-GGATTCTATCTGCATCTCAGGAG-3’) with probe (5’-/56-FAM/CCCCGTGGA/ZEN/ AGACACCATCATTGG/3IABkFQ/-3’).

### Statistical analysis

Statistics tests were performed in GraphPad Prism version 10.0 (GraphPad Software). Unless indicated otherwise, values are presented as mean ± standard error of the mean (SEM). Areas of Iba1^+^ cell bodies were calculated using Halo 4.0 (Indica Labs). Statistical differences between the groups were compared using one-way ANOVA or two-way ANOVA with Tukey’s multiple comparison test for multiple groups. *P* < 0.05 was considered statistically significant.

## Results

### Expression of ATXN1 with an expanded polyQ tract does not affect viability and function of spinal cord oligodendrocytes, astrocytes, and motor neurons

**Figure 1:**
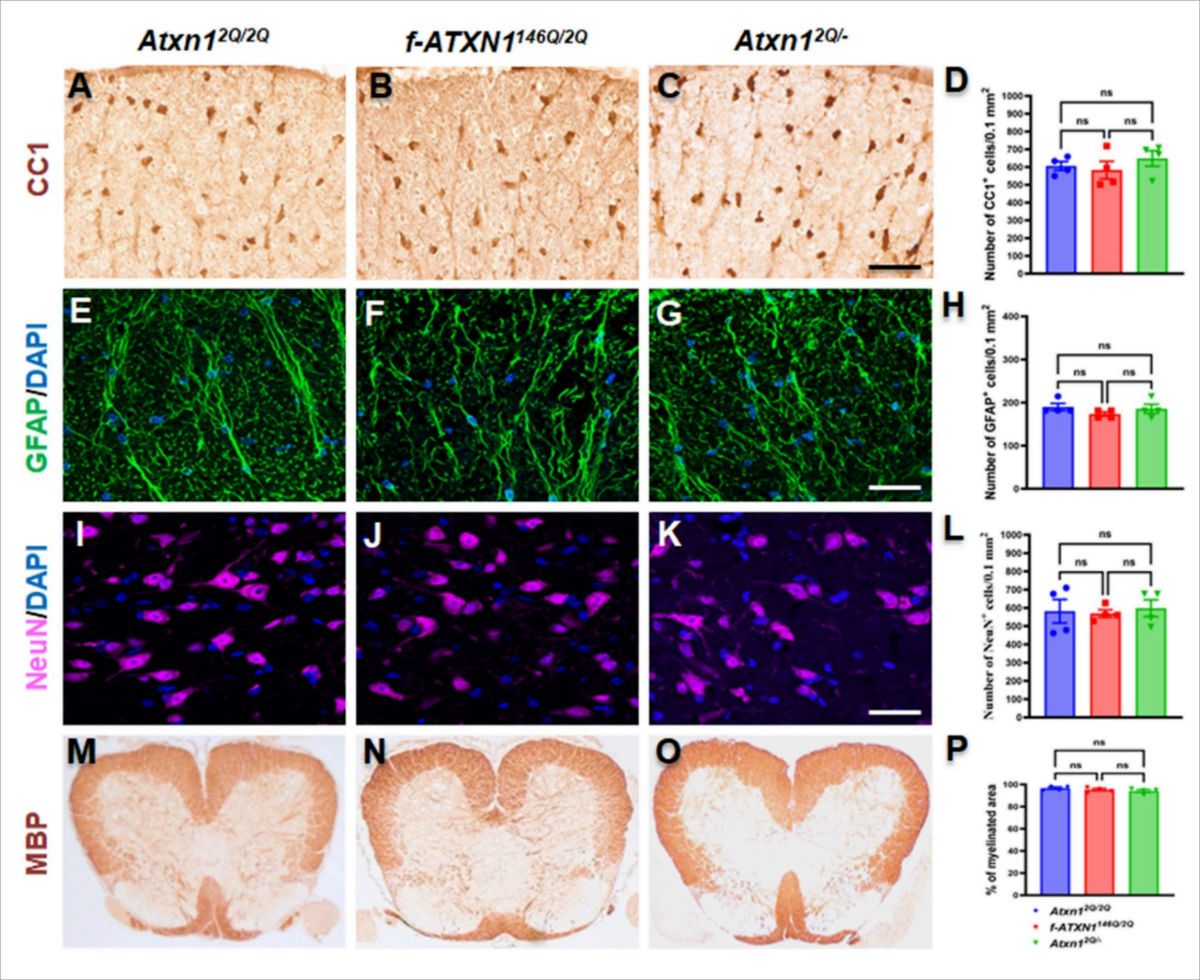
Nerve and glial cell viability and myelination are not altered at the age of 8 weeks in *f-ATXN1*^*146Q/2Q*^ and *Atxn1*^*2Q/*−^ mice. (**A–D)** CC1 IHC (DAB stain) showed a comparable number of oligodendrocytes in the lumbar spinal cord of WT mice, *f-ATXN1*^*146Q/2Q*^ mice, and *Atxn1*^*2Q/*−^ mice. (**E–H**) GFAP stain showed no differences in astrocytes (green) number, with nuclear counterstain DAPI (Blue), at the white matter of the lumbar spinal cord among the groups. (**I–L)** NeuN stain showed similar number of motor neurons (purple), with nuclear counterstain DAPI (Blue), in the grey matter of the lumbar spinal cord among all groups of mice. MBP IHC showed a comparable degree of myelination in lumbar spinal cord of the WT mice, *f-ATXN1*^*146Q/2Q*^ and *Atxn1*^*2Q/*−^ mice (**M–P**). N = 4 animals each. Scale bars: 40 μm (**A–C**, **E–G**, **I–K**). Statistical analyses were done with a one-way ANOVA with a Tukey’s multiple comparison test. Error bars represent SEM; ns, not significant.

Spinocerebellar ataxia type 1 (SCA1) is caused by expansion of glutamine(Q) encoding CAG repeats in *ATXN1* that results in a toxic gain of ATXN1 function^23^. We first examined whether cells involved in MS are altered in untreated *Atxn1*^*2Q/2Q*^ (WT) mice, *f-ATXN1*^*146Q/2Q*^ (*Atxn1* heterozygous knock-in) mice, and *Atxn1*^*2Q/*−^ (*Atxn1* heterozygous knock-out) mice on a C57BL/6J background. To characterize the mice, we collected the lumbar spinal cord at 8 weeks of age. The spinal cord, more specifically the lumbar spinal cord, is a critical region for EAE mediated lesion^24^. We performed DAB staining with CC1, an antibody marker for oligodendrocytes, and found comparable numbers of oligodendrocytes in the white matter of lumbar spinal cord of WT, *f-ATXN1*^*146Q/2Q*^, and *Atxn1*^*2Q/*−^ mice (Fig. 1A, B, C, D). Immunostaining for GFAP (a marker for astrocytes) (Fig. 1E, F, G, H) and NeuN (a marker for motor neurons) (Fig. 1I, J, K, L) didn’t show any differences in their numbers in the white matter and grey matter respectively among all the three groups of mice. DAB staining of myelin basic protein (MBP), showed similar degree of myelination in the white matter of lumbar spinal cord (Fig. 1M, N, O, P), in the cerebellum and brain stem (Supplementary Fig. 1A, B, C) and in the corpus callosum (Supplementary Fig. 1D, E, F). Taken together, these data indicate that neither ATXN1 loss nor ATXN1 with an expanded polyQ impacts neuronal and glial viability, gliosis or myelination in the lumbar spinal cord of 8-week-old mice under normal physiological conditions.

### ATXN1 loss-of-function exacerbates EAE disease severity

**Figure 2:**
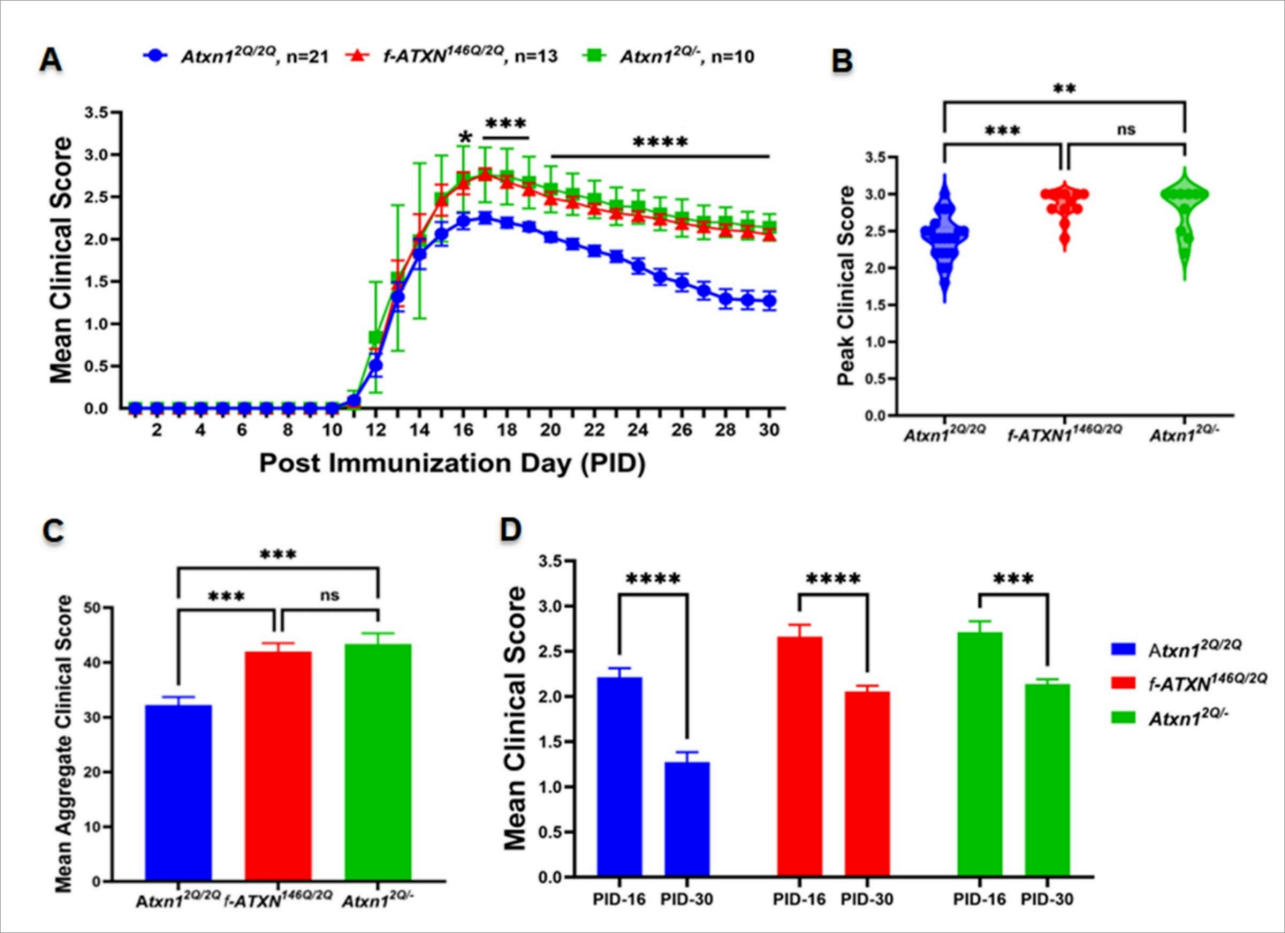
EAE severity is enhanced in *f-ATXN1*^*146Q/2Q*^ and *Atxn1*^*2Q/*−^ mice. (**A)** Mean EAE clinical scores showed an identical more severe disease course in *f-ATXN1*^*146Q/2Q*^ and *Atxn1*^*2Q/*−^ mice compared to WT control mice. (**B**) Peak EAE clinical score for individual mice. (**C**) The mean aggregate EAE clinical score was higher in *f-ATXN1*^*146Q/2Q*^ and *Atxn1*^*2Q/*−^ mice compared to WT control mice. (**D**) Mean EAE clinical scores PID-16 and PID-30 for WT (N = 21), *f-ATXN1*^*146Q/2Q*^ (N = 13), and *Atxn1*^*2Q/*−^ mice (N = 10). Error bars represent SEM. Differences between scores on each day were assessed by two-way ANOVA with Tukey’s multiple comparison test (**A, D**) and one-way ANOVA with Tukey’s multiple comparison test (**B, C**). * (knock-in vs. wildtype; heterozygous vs. wildtype); *p≤ 0.05, **p≤ 0.01, ***p≤ 0.001, ****p≤ 0.0001, ns, not significant.

To examine the role of ATXN1 in EAE, we immunized 8-week-old WT, *f-ATXN1*^*146Q/2Q*^, and *Atxn1*^*2Q/*−^ female mice, with myelin oligodendrocyte glycoprotein (MOG) peptide 35 to 55 (MOG_35–55_) to induce experimental autoimmune encephalomyelitis (EAE). While all the mice showed impairments consistent with EAE, *f-ATXN1*^*146Q/2Q*^ and *Atxn1*^*2Q/*−^ mice had greater clinical scores compared to WT controls indicating exacerbated disease severity (Fig. 2A). Importantly, compared to WT littermates *f-ATXN1*^*146Q/2Q*^ and *Atxn1*^*2Q/*−^ mice also had impaired recovery (Fig. 2A). Although there were no differences in the onset and early disease progression, *f-ATXN1*^*146Q/2Q*^ and *Atxn1*^*2Q/*−^ mice exhibited a higher peak score (Fig. 2B) and higher mean aggregate score (Fig. 2C) than WT mice. Disease recovery from peak (PID-16) to remission (PID-30) was attenuated in *f-ATXN1*^*146Q/2Q*^ and *Atxn1*^*2Q/*−^ mice when compared to WT mice (Fig. 2D). We observed no differences in disease onset, disease progression, and remission between *f-ATXN1*^*146Q/2Q*^ and *Atxn1*^*2Q/*−^ mice (Fig. 2A, B, C), indicating that *ATXN1* genetic modification by polyQ expansion and by knock-out has similar effects on EAE disease course.

### ATXN1 loss-of-function enhances oligodendrocyte loss and demyelination during EAE

**Figure 3:**
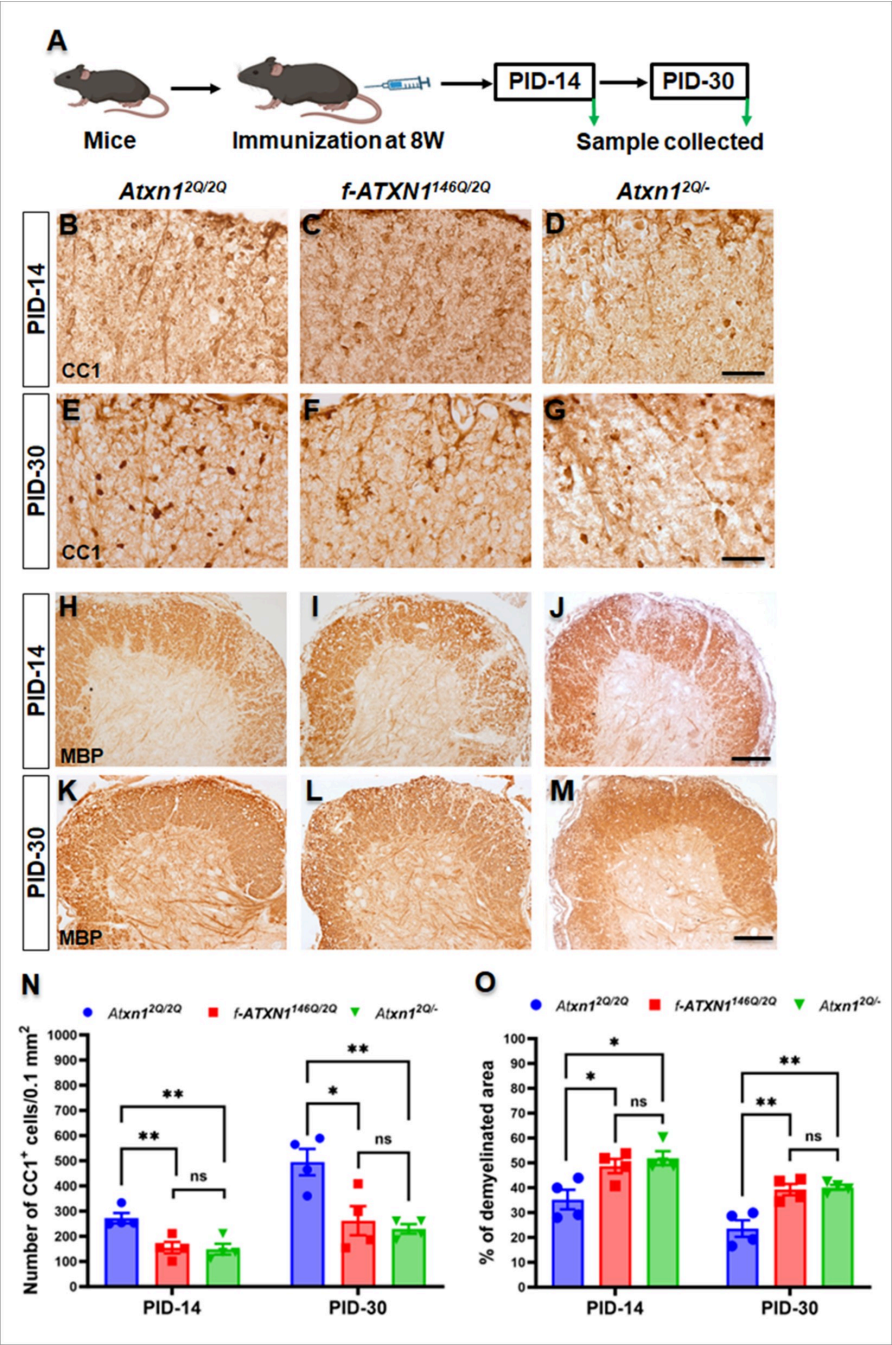
EAE-induced oligodendrocyte loss and demyelination are more severe in *f-ATXN1*^*146Q/2Q*^ and *Atxn1*^*2Q/*−^ mice. (**A**) Diagram depicting the sample collection protocol. (**B–G, N**) DAB staining of the CC1 in the lumbar spinal cord showed significantly higher loss of oligodendrocytes in *f-ATXN1*^*146Q/2Q*^ and *Atxn1*^*2Q/*−^ mice during peak disease at PID-14 (**B–D**) and during remission at PID-30 (**E–G**), as with WT mice. There were no differences in the level of oligodendrocyte loss between *f-ATXN1*^*146Q/2Q*^ mice and *Atxn1*^*2Q/*−^ mice at PID-14 and PID-30. (**H–M, O**) DAB staining of the MBP showed that the percentage of demyelinated area in the lumbar spinal cord was significantly increased in *f-ATXN1*^*146Q/2Q*^ mice and *Atxn1*^*2Q/*−^ mice compared to WT mice at PID-14 (**H–J**) and PID-30 (**K–M**). No significant differences in the degree of demyelination in the lumbar spinal cord were observed between *f-ATXN1*^*146Q/2Q*^ and *Atxn1*^*2Q/*−^ mice at PID-14 and PID-30. N = 4 animals each. Scale bars: 40 μm (**B–G**) and 100 μm (**H–M**). Statistical analyses were done with one-way ANOVA with Tukey’s multiple comparison test. Error bars represent SEM; *p≤ 0.05, **p≤ 0.01, ns, not significant.

Previous studies show that loss of PERK signaling in oligodendrocytes increases susceptibility to inflammation, resulting in exacerbation of demyelination in EAE^25^. Additionally, ATF6α deficiencies linked to exacerbated oligodendrocyte loss during EAE, highlighting the importance of specific signaling pathways in maintaining oligodendrocyte viability and myelin integrity^21^. Furthermore, demyelination and oligodendrocyte loss are features of EAE lesions^26^. Inflammatory demyelination induces axonal injury and neuronal apoptosis, emphasizing the interconnection of these processes in neuroinflammatory conditions^27^. To assess how ATXN1 loss impacts oligodendrocyte number and demyelination, 8-week-old mice were immunized with MOG_35–55_ and tissues collected from lumbar spinal cord at PID-14 and PID-30 (Fig. 3A). DAB staining of CC1 revealed that few oligodendrocytes remained in the lesions of lumbar spinal cord of each genotype, whereas *f-ATXN1*^*146Q/2Q*^ and *Atxn1*^*2Q/*−^ mice showed significantly higher reductions in oligodendrocyte numbers compared to WT mice at peak disease progression at PID-14 (Fig. 3B, C, D, N). During the disease remission period at PID-30, the numbers of newly generated oligodendrocytes were reduced in the lumbar spinal cord of *f-ATXN1*^*146Q/2Q*^ and *Atxn1*^*2Q/*−^ mice compared to WT mice (Fig. 3E, F, G, N). Furthermore, quantitative analysis of MBP IHC showed around 50% of the white matter of lumbar spinal cord of *f-ATXN1*^*146Q/2Q*^ and *Atxn1*^*2Q/*−^ mice was demyelinated at PID-14 which was significantly higher than in WT mice (< 35%) (Fig. 3H, I, J, O). Despite remyelination in the recovery period at PID-30, the demyelinated area continued to be higher in the white matter of lumbar spinal cord of *f-ATXN1*^*146Q/2Q*^ and *Atxn1*^*2Q/*−^ mice than WT mice (Fig. 3K, L, M, O). Interestingly, there were no differences in oligodendrocyte loss and the degree of demyelination between *f-ATXN1*^*146Q/2Q*^ and *Atxn1*^*2Q/*−^ mice at PID-14 and PID-30 (Fig. 3N, O).

### ATXN1 deficiency promotes axonal degeneration during EAE

**Figure 4:**
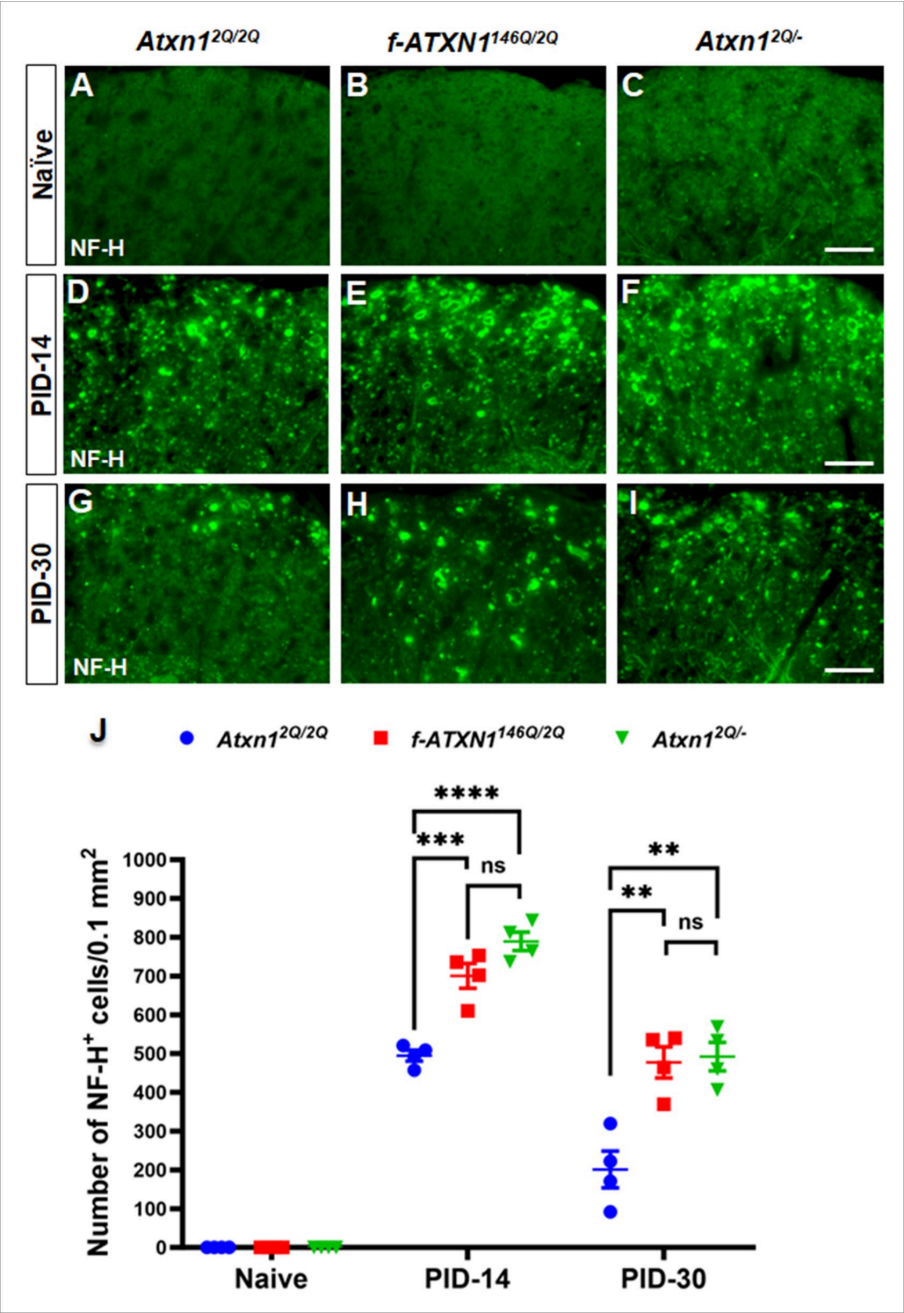
EAE-induced axon degeneration is enhanced in *f-ATXN1*^*146Q/2Q*^ and *Atxn1*^*2Q/*−^ mice. (**A–C, J**) Immunofluorescence staining of the NF-H for degenerating axons in the lumbar spinal cord showed that no axon degeneration was found in Naïve mice at age of 8 weeks (**A–C**). (**D–F, J**) Immunofluorescence staining of NF-H in lumbar spinal cord displayed significantly higher number of degenerating axons in *f-ATXN1*^*146Q/2Q*^ and *Atxn1*^*2Q/*−^ mice during accelerated disease progression at PID-14 (**D–F**) and during remission at PID-30 (**G–I**) compared to WT mice. (**J**) *f-ATXN1*^*146Q/2Q*^ and *Atxn1*^*2Q/*−^ mice showed comparable number of degenerating axons at both time points. N = 4 animals each. Scale bars: 40 μm. Statistical analyses were done with one-way ANOVA with Tukey’s multiple comparison test. Error bars represent SEM; *p≤ 0.05, **p≤ 0.01, ***p≤ 0.001, ****p≤ 0.001, ns, not significant.

Axonal degeneration is a critical aspect of neurological deficits in autoimmune conditions such as EAE and MS. Studies show that axonal degeneration contributes significantly to the development of non-remitting neurological deficits and disability in MS^28^. Axonal degeneration within spinal cord lesions of EAE animals has been well characterized^25^. Furthermore, axonal degeneration is associated with the development of neurological disability in MS and EAE^29^. Therefore, we performed immunofluorescence staining of the non-phosphorylated neurofilament-H (NF-H, previously identified as SMI-32), a marker for degenerating axons, in the lumbar spinal cord of each genotype of naïve 8-week-old mice, PID-14, and PID-30. As expected, no degenerating axons were seen in the white matter of lumbar spinal cord in 8-week-old under normal conditions (Fig. 4A, B, C, J). There was substantial axonal degeneration in the white matter of lumbar spinal cord at PID-14 and the number of degenerating axons was significantly higher in *f-ATXN1*^*146Q/2Q*^ and *Atxn1*^*2Q/*−^ mice than WT mice. We found no differences in the number of degenerating axons between *f-ATXN1*^*146Q/2Q*^ and *Atxn1*^*2Q/*−^ mice (Fig. 4D, E, F, J). With recovery the number of degenerating axons was greatly reduced in the white matter of lumbar spinal cord of WT mice, but significantly less so in *f-ATXN1*^*146Q/2Q*^ and *Atxn1*^*2Q/*−^ mice. The number of degenerating axons was comparable in *f-ATXN1*^*146Q/2Q*^ and *Atxn1*^*2Q/*−^ mice at PID-30 (Fig. 4G, H, I, J). These data suggest that the normal function of ATXN1 supports axonal survivability upon EAE challenges.

### Mutant ATXN1-mediated activation of astrocytes plays a dual role during EAE

**Figure 5:**
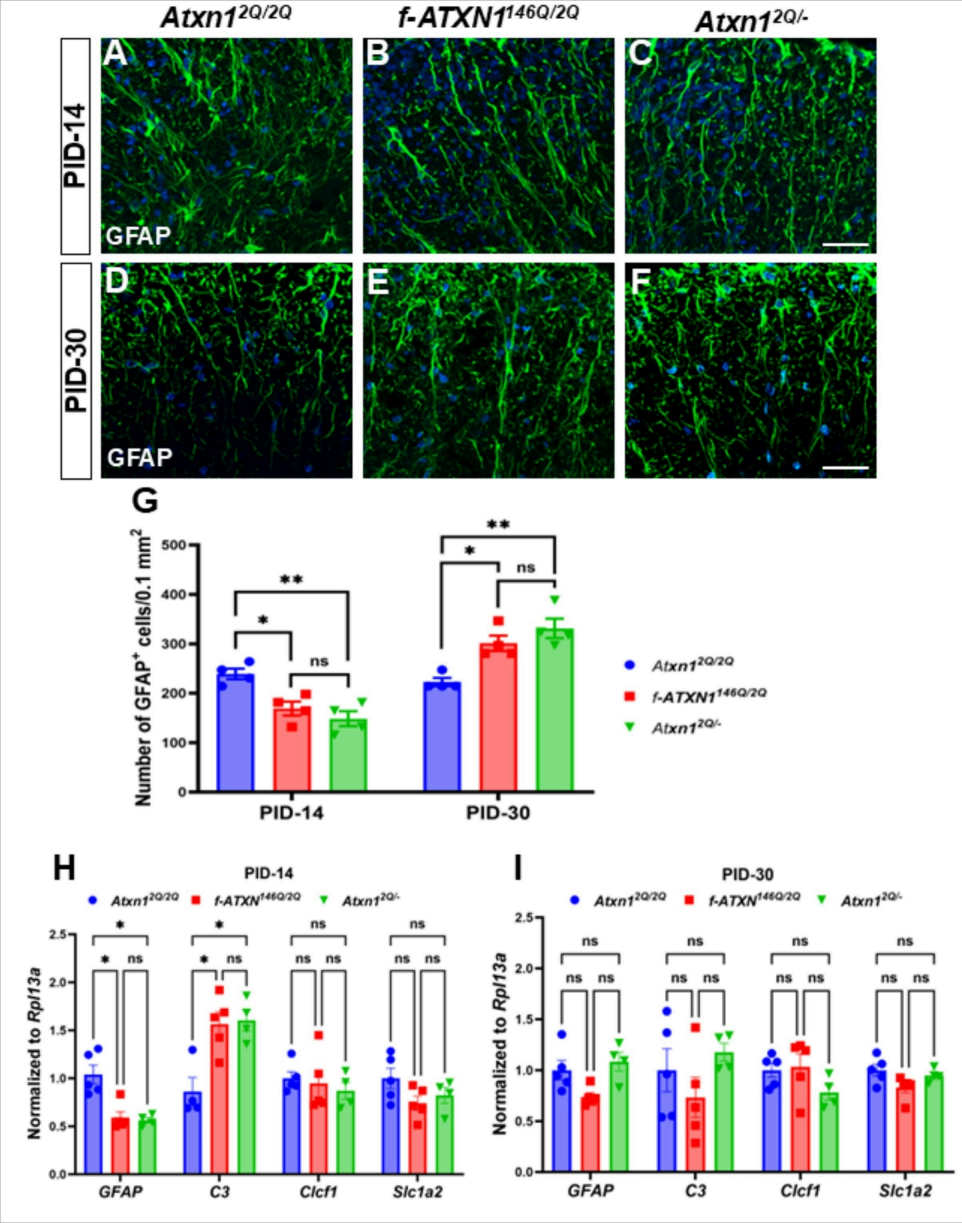
Alteration in EAE-induced reactive astrocytes at early and late Post Immunization Days. GFAP immunofluorescence staining (green) and nuclear counterstain, DAPI (blue) showed ablation of reactive astrocytes in the white matter of lumbar spinal cord of *f-ATXN1*^*146Q/2Q*^ and *Atxn1*^*2Q/*−^ mice compare to WT controls in the acute phage of disease progression at PID-14 (**A–C, G**). In the chronic phage of diseases progression at PID-30 (**D–f, G**), the elevated number of reactive astrocytes were counted in the white matter of lumbar spinal cord of both *f-ATXN1*^*146Q/2Q*^ and *Atxn1*^*2Q/*−^ mice compared to WT controls. N = 4 animals each. Scale bars: 40 μm. RT-qPCR analysis showing the lower levels of *GFAP* and higher levels of *C3* in the lumbar spinal cord of *f-ATXN1*^*146Q/2Q*^ and *Atxn1*^*2Q/*−^ mice compared to WT controls at PID-14 (**H**), while levels were comparable among three groups at PID-30 (**I**). No significant differences of *Clcf1* and *Slc1a2* expressions were observed in the lumbar spinal cord of WT, *f-ATXN1*^*146Q/2Q*^, and *Atxn1*^*2Q/*−^ mice at either PID-14 (**H**) or PID-30 (**I**). N = 4**–**5 animals each. Statistical analyses were done with one-way ANOVA with Tukey’s multiple comparison test. Error bars represent SEM; *p≤ 0.05, **p≤ 0.01, ns, not significant.

Reactive astrocytes play a crucial role in the pathophysiology of EAE and respond to insults by undergoing a process known as reactive astrogliosis, which involves activation, hypertrophy, and proliferation^30^. Reactive astrocytes can have both beneficial and detrimental effects in EAE. As reactive astrocytes may serve to protect the CNS from injury by releasing growth factors^31^, inhibition of reactive astrogliosis can lead to more severe inflammation and clinical symptoms^32^. Immunofluorescence staining of GFAP revealed a reduced number of reactive astrocytes in the white matter of lumbar spinal cord at the time of severe disease progression at PID-14 (Fig. 5A, B, C, G) and increased number of reactive astrocytes at the time of remission at PID-30 (Fig. 5D, E, F, G) in *f-ATXN1*^*146Q/2Q*^ and *Atxn1*^2Q/−^ mice compared to WT mice. Quantitative analysis showed that the severe inflammation during EAE has a similar effect on the number of reactive astrocytes between *f-ATXN1*^*146Q/2Q*^ and *Atxn1*^*2Q/*−^ mice (Fig. 5G). We performed RT-qPCR to investigate whether the astrocytes are A1 neurotoxic (*C3*) or A2 neuroprotective (*Clcf1* and *Slc1a2*). The *GFAP* mRNA level was significantly reduced in *f-ATXN1*^*146Q/2Q*^ and *Atxn1*^*2Q/*−^ mice compared to WT mice at PID-14 but no significant differences were detected at PID-30 (Fig. 5H, I). The expression of *C3* was higher in *f-ATXN1*^*146Q/2Q*^ and *Atxn1*^*2Q/*−^ mice than in WT mice at PID-14 but unchanged at PID-30 (Fig. 5H, I). No significant changes were observed in the expression of A2 markers *Clcf1* and *Slc1a2* among the three groups of mice at either PID-14 or PID-30 (Fig. 5H, I). These data reveal that the loss of ATXN1 activates neurotoxic astrocytes at PID-14 of EAE. In contrast, astrocytes no longer show signs of activation at PID-30 of EAE.

### Elevated numbers of infiltrated T cells and macrophages/microglia in the lesions of the lumbar spinal cord (white matter) upon EAE

**Figure 6:**
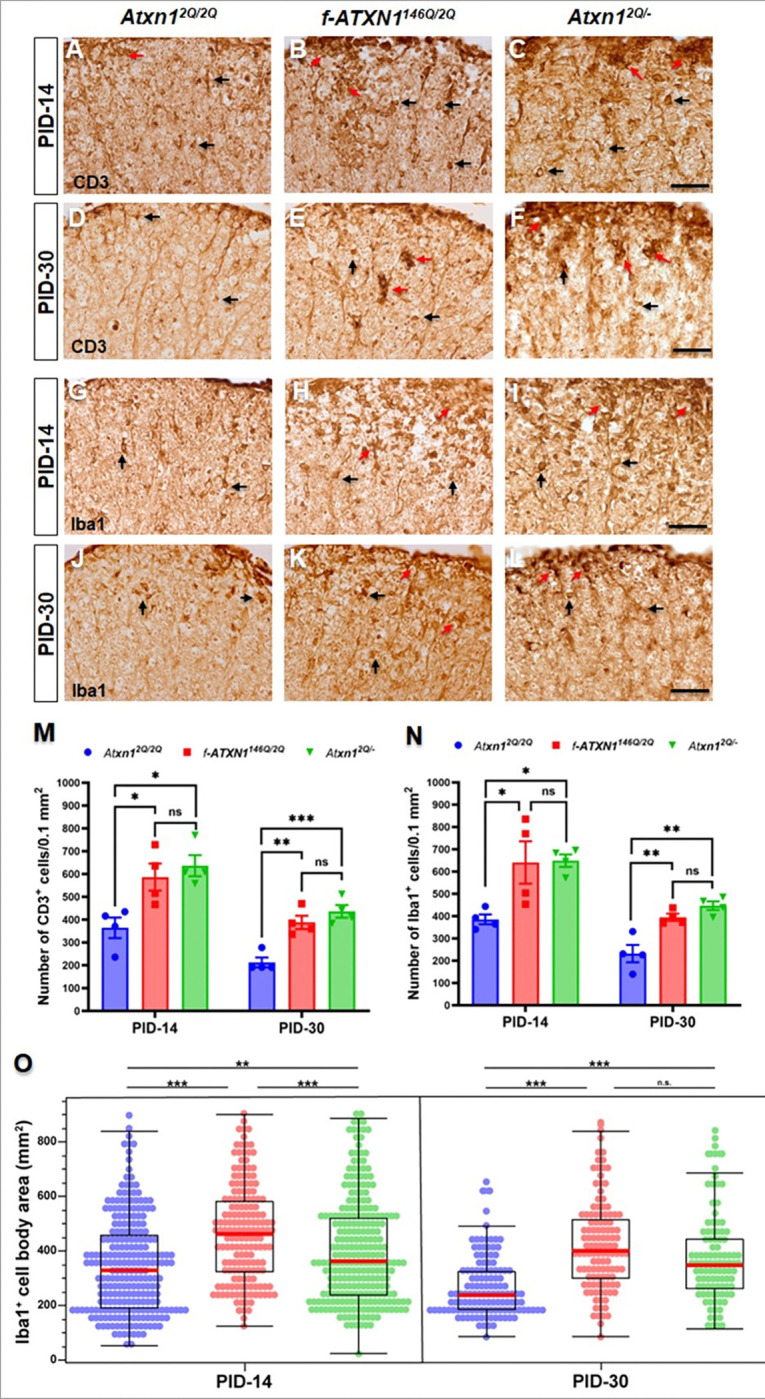
Infiltration of inflammatory T cells and macrophages/microglia in the white matter of EAE-induced lumbar spinal cord lesions at early and late Post Immunization Days. (**A–F, M**) DAB staining of CD3 (T cells) showed higher number of T cells at PID-14 (**A–C**) as well as at PID-30 (**D–F**) in the lumbar spinal cord of *f-ATXN1*^*146Q/2Q*^ and *Atxn1*^*2Q/*−^ mice compared to WT controls. No significant differences were seen between the lumbar spinal cord of *f-ATXN1*^*146Q/2Q*^ and *Atxn1*^*2Q/*−^ mice. (**G–J, N**) DAB staining of Iba1 (macrophages/microglia) showed a higher number of macrophages/microglia at PID-14 (**G–I**) as well as at PID-30 (**J–L**) in the lumbar spinal cord of *f-ATXN1*^*146Q/2Q*^ and *Atxn1*^*2Q/*−^ mice compared to WT controls. No significant differences were seen between the lumbar spinal cord of *f-ATXN1*^*146Q/2Q*^ and *Atxn1*^*2Q/*−^ mice. (**G–J, O**) Morphology analysis showed a higher number of hypertrophic and/or amoeboid macrophages/microglia at PID-14 as well as at PID-30 in the lumbar spinal cord of *f-ATXN1*^*146Q/2Q*^ and *Atxn1*^*2Q/*−^ mice compared to WT controls (**O**). A significantly higher number of hypertrophic and/or amoeboid macrophages/microglia were observed at PID-14, but their numbers were comparable between the *f-ATXN1*^*146Q/2Q*^ and *Atxn1*^*2Q/*−^ mice at PID-30 (**O**). N = 4 animals each. Scale bars: 40 μm. Black arrows indicate single cells, and red arrows indicate clusters of activated T cells and macrophages/microglia. Statistical analyses were done with one-way ANOVA with Tukey’s multiple comparison test. Error bars represent SEM; *p≤ 0.05, **p≤ 0.01, ***p≤ 0.001, ns, not significant.

The initiation of EAE is characterized by the peripheral formation of myelin-reactive encephalitogenic T lymphocytes that migrate to the CNS, where they trigger neuroinflammation in collaboration with microglia and infiltrated macrophages^33^. The intricate balance between effector T cells and regulatory T cell subsets influences the development and progression of EAE, highlighting the complexity of T cell responses in autoimmune demyelinating diseases. To assess the number of infiltrated T cells in the lesions of lumbar spinal cord upon EAE, we performed DAB staining of CD3 (a marker for T cells) at PID-14 and PID-30. Higher numbers of activated T cells and a cluster of activated T cells were seen at PID-14 (Fig. 6A, B, C, M) as well as at PID-30 (Fig. 6D, E. F, M) in the lumbar spinal cord of *f-ATXN1*^*146Q/2Q*^ and *Atxn1*^*2Q/*−^ mice than WT mice.

DAB staining of Iba1 (a macrophages/microglia marker) showed significantly higher numbers of macrophages/microglia at the inflammatory lesion in the lumbar spinal cord of *f-ATXN1*^*146Q/2Q*^ and *Atxn1*^*2Q/*−^ mice compared to WT mice at PID-14 (Fig. 6G, H, I, N) and PID-30 (Fig. 6J, K, L, N). To determine morphological changes of macrophages/microglia we further analyzed the DAB staining of Iba1 by parametric approach (Supplementary Fig. 2). Morphology analysis showed the higher number of hypertrophic and/or amoeboid macrophages/microglia in the lumbar spinal cord of *f-ATXN1*^*146Q/2Q*^ and *Atxn1*^*2Q/*−^ mice compared to WT mice at PID-14 (Fig. 6G, H, I, O) and PID-30 (Fig. 6J, K, L, O). A significantly higher number of hypertrophic and/or amoeboid macrophages/microglia were observed at PID-14, but their numbers were comparable between the *f-ATXN1*^*146Q/2Q*^ and *Atxn1*^*2Q/*−^ mice at PID-30 (Fig. 6G, H, I, O). The elevated expression of hypertrophic and/or amoeboid macrophages/microglia at PID-14 in the lumbar spinal cord of *f-ATXN1*^*146Q/2Q*^ mice compared to *Atxn1*^*2Q/*−^ mice may be due to expanded polyQ mediated activation. These results indicate that loss of ATXN1 exacerbates immune activation in EAE.

### Altered immune cytokine gene expression in the lumbar spinal cord in response to inflammation during EAE

**Figure 7:**
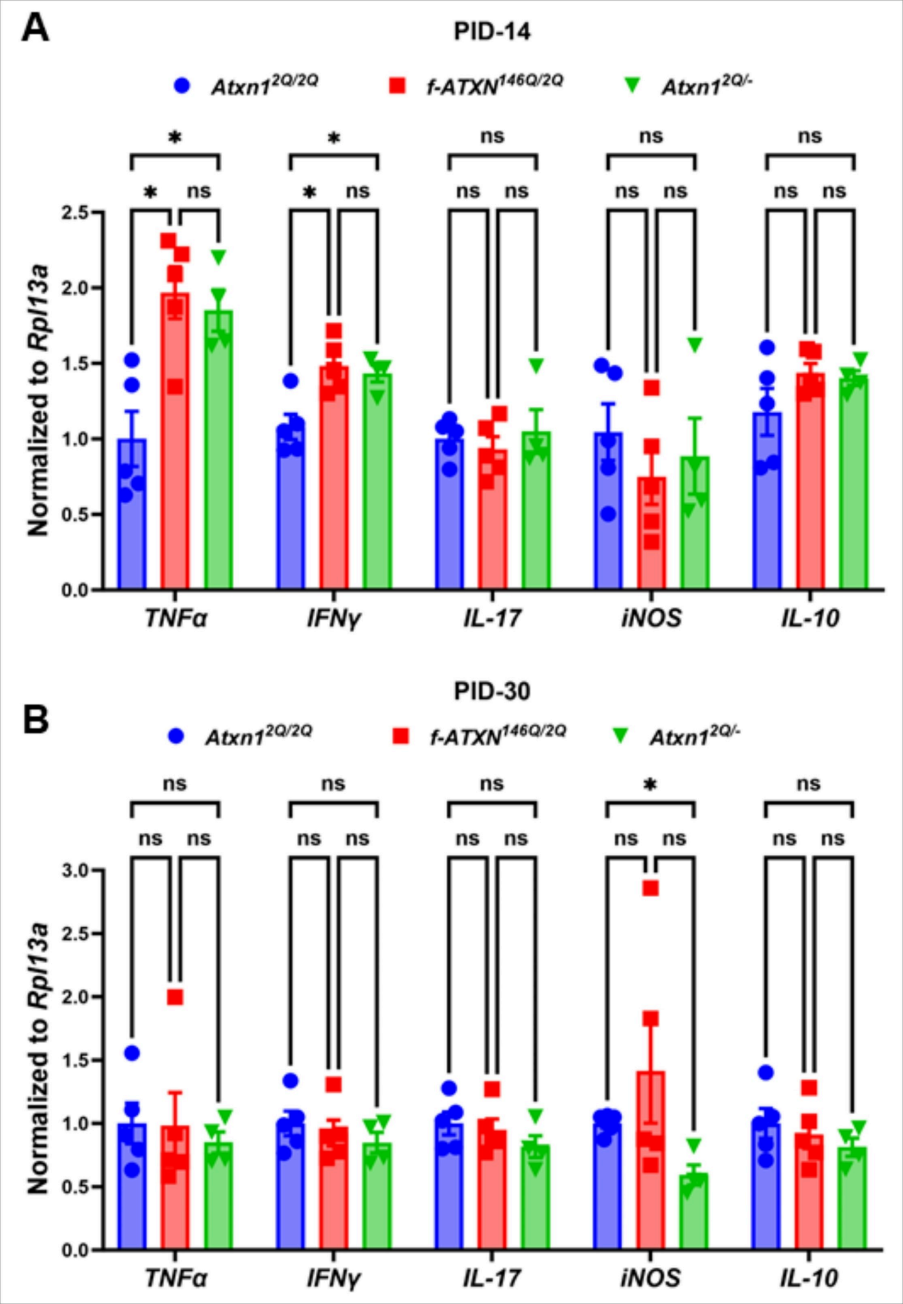
Inflammatory cytokines at the lesion of lumbar spinal cord during EAE. RT-qPCR analysis showing the higher levels of *TNFα* and *IFNγ* in the lumbar spinal cord of *f-ATXN1*^*146Q/2Q*^ and *Atxn1*^*2Q/*−^ mice compared to WT controls at PID-14 (**A**) but comparable at PID-30 (**B**). No significant differences of *TNFα* and *IFNγ* levels were seen between the lumbar spinal cord of *f*-*ATXN1*^*146Q/2Q*^ and *Atxn1*^*2Q/*−^ mice (**A, B**). No significant differences of *IL-17* and *IL-10* levels were seen among the lumbar spinal cord of WT, *f-ATXN1*^*146Q/2Q*^ and *Atxn1*^*2Q/*−^ mice at PID-14 (**A**) and PID-30 (**B**). At PID-30, *Atxn1*^*2Q/*−^ mice showed lower levels of *iNOS* compared to WT controls, whereas no significant differences were observed among all three groups of mice at PID-14 (**A**, **B**). N = 5 animals each. Statistical analyses were done with one-way ANOVA with Tukey’s multiple comparison test. Error bars represent SEM; *p≤ 0.05, ns, not significant.

The expression of various cytokines during EAE reflects the complex interplay between pro-inflammatory and anti-inflammatory responses that dictate disease progression and severity. RT-qPCR analysis showed higher expression of *TNFα* and *IFNγ* in the lumbar spinal cord of *f-ATXN1*^*146Q/2Q*^ and *Atxn1*^*2Q/*−^ mice compared to WT mice at PID-14 (Fig. 7A). No significant differences in the expression of *TNFα* and *IFNγ* were seen in the chronic phase of the EAE at PID-30 (Fig. 7B). Similarly, no changes in *IL-17* and *IL-10* levels were observed among all three groups of mice at PID-14 and PID-30 (Fig. 7A, B). Previous research showed that the role of *iNOS* may shift, potentially influencing the resolution of inflammation and tissue repair processes in chronic phases^34,35^. Consistent with this, our RT-qPCR showed the lower levels of *iNOS* in lumbar spinal cord of *Atxn1*^*2Q/*−^ compared to WT control at PID-30 (Fig. 7B), but no significant differences at PID-14 among these three groups of mice (Fig. 7A). Taken together, these data indicate that mutant ATXN1 alters inflammation in the CNS of mice during EAE more specifically in the acute phase of the disease.

## Discussion

Genome-wide genomic screens link the *ATXN1* locus with an increased risk of developing MS^9^. In the present study, using two different genetic modifications of *Atxn1* in mice, we demonstrated that haploinsufficiency of *Atxn1* and expansion of the CAG repeat exacerbate clinical EAE symptoms as well as underlying pathology, including axonal degeneration, oligodendrocyte loss and demyelination, and immune activation during acute and chronic phases of the disease progression. Thus, expansion of the polyQ tract in ATXN1 impacts EAE severity through a loss-of-function mechanism. Importantly, neither of these ATXN1 genetic modifications caused cellular loss nor demyelination in the spinal cord of mice in absence of EAE.

ATXN1 expression is controlled by hypomethylation at specific genomic sites within the ATXN1 sequence in B cells at clinical onset of the disease^10^. This regulation suggests that ATXN1 may enhance B cell function, which is crucial since B cells contribute to the autoimmune response observed in MS. The ability of ATXN1 to modulate B cell activity can influence the production of antibodies and cytokines, thereby affecting the overall inflammatory response in the central nervous system (CNS). Moreover, ATXN1 has been shown to regulate the signaling pathways involved in B cell receptor (BCR) signaling. Ma and Didonna reported that ATXN1 affects the extracellular signal-regulated kinase (ERK) and signal transducer and activator of transcription (STAT) pathways, which are critical for B cell activation and proliferation^8^. By fine-tuning these signaling pathways, WT ATXN1 may help maintain a balance between pro-inflammatory and anti-inflammatory responses, potentially mitigating MS severity. Previous studies highlighted that CIC deficiency leads to upregulation of TNF-α in liver macrophages, suggesting that CIC may similarly regulate cytokine expression in other immune contexts, including EAE^36^. This regulation is crucial, as TNF-α is known to enhance the activation of T cells and the recruitment of inflammatory cells to the central nervous system (CNS) during EAE^37^.

In the context of MS, ATXN1 was found to regulate B cell function, impacting the severity of autoimmune experimental encephalomyelitis^7^. The ablation of ATXN1 in B cells results in aberrant expression of key molecules involved in proinflammatory T cell differentiation, suggesting a role for ATXN1 in modulating immune responses^7^. Consistent with these observations, our data showed that the disease severity upon EAE is associated with the recruitment of a higher number of activated T cells and macrophage/microglia to the lesion sites. TNFα is known to be upregulated in EAE and contributes significantly to initiation and amplification of the immune response within the CNS. TNFα promotes the recruitment and activation of various immune cells, including macrophages and T cells, which infiltrate the CNS and exacerbate inflammation^38^. The presence of TNFα is associated with increased blood-brain barrier permeability, facilitating immune cell entry into the CNS and leading to further tissue damage^39^. Similarly, IFNγ plays a crucial role in the pathogenesis of EAE. IFNγ enhances macrophage and microglia activation, leading to increased production of pro-inflammatory cytokines and mediators, including TNFα itself^40^. IFNγ is also involved in the differentiation of naive T cells into Th1 cells, thereby perpetuating the inflammatory cycle^41^. Research has demonstrated that mice deficient in IFNγ exhibit increased susceptibility to EAE, highlighting its protective role in modulating the immune response^42^. Consequently, the elevated production of inflammatory cytokines like TNFα and IFNγ in the CNS during EAE are notable. Although IFNγ and TNFα play important roles in the first attack, they are not major contributors to relapse^42^, which is consistent with our findings.

Furthermore, studies show that mutant ATXN1 mediated activation of astrocytes and microglia at early ages in SCA1 models lead to a pro-inflammatory environment, that may closely relate to neuronal dysfunction and damage^5,43^. Activation of neurotoxic astrocytes at an early stage (PID-14) of disease contributes to increase severity, whereas the absence of neurotoxic astrocytes activation at a later stage (PID-30) presumably allows the initiation of recovery from disease-related damage. The interaction between the activated glial cells and CD3-positive T cells in the cerebellum of SCA1 mice reflects the involvement of T cells in the neuroinflammatory response associated with the disease, indicating a potential link between immune activation and the degeneration of Purkinje cells.

Additionally, GFAP expressions can differ during early phases and in remitting EAE. GFAP levels were significantly elevated, reflecting broad activation of astrocytes in response to inflammatory cues and tissue damage^44^. Studies have also shown reduced levels of GFAP in spinal cord at the acute phase of disease during EAE^45,46^ which is consistent with our findings. During EAE progression, microglia become activated and release various pro-inflammatory cytokines, including IL-1α and TNF-α, contributing to the synthesis of C3 in astrocytes and enhancing neurotoxic activity^47^. A complex interplay between microglia and astrocytes where microglial activity initiates the release of C3 and induces increased expression in neighboring astrocytes, creating a feedback loop that may exacerbate inflammatory responses in EAE. Therefore, severe phagocytic activity of microglia might also contribute to reduced levels of astrocytes (both cells and mRNA) at the acute phase (PID-14) of EAE (Fig. 5) in the mutant mice. The increased levels of GFAP at the white matter of lumbar spinal cord, despite similar mRNA levels in the whole lumbar spinal cord at PID-30, may be due to the differential responses of white and grey matter to EAE-induced inflammation.

Intriguingly, we found that loss of ATXN1 caused a reduced number of reactive astrocytes during acute EAE disease progression in *Atxn1*^*2Q/*−^ mice while a higher number of reactive astrocytes was present in the white matter of lumbar spinal cord of these mice during remission of the disease. Studies show that depletion of reactive astrocytes during the acute phase is associated with worsening EAE outcomes^48^. Reactive astrocytes orchestrate inflammatory responses of resident and peripheral immune cells in the CNS during EAE^30^. They can suppress remyelination and contribute to the inflammatory milieu by releasing cytokines and chemokines^30^. A common aspect of neurodegenerative disease is neuroinflammation^4^. In particular, signs of neuroinflammation are detected in mouse models of SCA3^6^ and SCA1^49^. We speculate that a loss-of-function in ATXN1 with an expanded polyQ tract has the ability to dampen immune responses might result in increased neuroinflammation during SCA1 pathogenesis.

In conclusion, loss-of-function of ATXN1 enhances severity of MS, potentially through its involvement in gene expression regulation, immune responses, and interactions with nuclear transport pathways. Further research into the specific mechanisms by which ATXN1 influences the pathogenesis of MS could provide valuable insights into novel therapeutic targets for this complex autoimmune inflammatory disease.

## Data Availability

No datasets were generated or analyzed during the current study.

## Abbreviations

Ataxin-1: (ATXN1)
SCA1: Spinocerebellar ataxia type 1
EAE: Experimental autoimmune encephalomyelitis
MS: Multiple sclerosis
MOG: Myelin oligodendrocyte glycoprotein
CNS: Central nervous system
PID: Post-immunization day
SC: Spinal cord
MBP: Myelin basic protein
GFAP: Glial fibrillary acidic protein
DAPI: 4′,6- diamidino-2-phenylindole
DAB: 3,3’-Diaminobenzidine
RT-qPCR: Real-time quantitative polymerase chain reaction
C3: Complement C3
Clcf1: Cardiotrophin-like cytokine factor 1
Slc1a2: Solute carrier family 1 member 2
TNFα: Tumor necrosis factor alpha
IFNγ: Interferon gamma
iNOS: Inducible nitric oxide synthase
IL-17: Interleukin 17
IL-10: Interleukin 10
ATF6: Activating transcription factor 6

## References

[1] Klement IA, Skinner PJ, Kaytor MD, Ataxin-1 Nuclear Localization and Aggregation: Role in Polyglutamine-Induced Disease in SCA1 Transgenic Mice. 10.1016/s0092-8674(00)81781-x 9778246

[2] Orr HT, Chung M yi, Banfi S, Expansion of an unstable trinucleotide CAG repeat in spinocerebellar ataxia type 1. Nat Genet. 1993;4(3):221–226. doi:10.1038/ng0793-221 8358429

[3] Globas C, Du Montcel ST, Baliko L, Early symptoms in spinocerebellar ataxia type 1, 2, 3, and 6. Movement Disorders. 2008;23(15):2232–2238. doi:10.1002/mds.22288 18759344

[4] Guzman-Martinez L, Maccioni RB, Andrade V, Navarrete LP, Pastor MG, Ramos-Escobar N. Neuroinflammation as a Common Feature of Neurodegenerative Disorders. Front Pharmacol. 2019;10:1008. doi:10.3389/fphar.2019.01008 31572186 PMC6751310

[5] Cvetanovic M, Ingram M, Orr H, Opal P. Early activation of microglia and astrocytes in mouse models of spinocerebellar ataxia type 1. Neuroscience. 2015;289:289–299. doi:10.1016/j.neuroscience.2015.01.003 25595967 PMC4344857

[6] Chiu YJ, Lin SA, Chen WL, Pathomechanism characterization and potential therapeutics identification for SCA3 targeting neuroinflammation. Aging. 2020;12(23):23619–23646. doi:10.18632/aging.103700 33196459 PMC7762503

[7] Didonna A, Canto Puig E, Ma Q, Ataxin-1 regulates B cell function and the severity of autoimmune experimental encephalomyelitis. Proc Natl Acad Sci USA. 2020;117(38):23742–23750. doi:10.1073/pnas.2003798117 32878998 PMC7519225

[8] Ma Q, Didonna A. The novel multiple sclerosis susceptibility gene ATXN1 regulates B cell receptor signaling in B-1a cells. Mol Brain. 2021;14(1):19. doi:10.1186/s13041-020-00715-0 33478569 PMC7819313

[9] Ma Q, Didonna A. Ataxin-1 controls the expression of specific noncoding RNAs in B cells upon autoimmune demyelination. Immunol Cell Biol. 2023;101(4):358–367. doi:10.1111/imcb.12622 36681886

[10] Ma Q, Oksenberg JR, Didonna A. Epigenetic control of ataxin-1 in multiple sclerosis. Ann Clin Transl Neurol. 2022;9(8):1186–1194. doi:10.1002/acn3.51618 35903875 PMC9380165

[11] Takeuchi H. Midkine and multiple sclerosis. British J Pharmacology. 2014;171(4):931–935. doi:10.1111/bph.12499 PMC3925032 24460675

[12] Calabrese M, Atzori M, Bernardi V, Cortical atrophy is relevant in multiple sclerosis at clinical onset. J Neurol. 2007;254(9):1212–1220. doi:10.1007/s00415-006-0503-6 17361339

[13] Meyer R, Weissert R, Diem R, Acute Neuronal Apoptosis in a Rat Model of Multiple Sclerosis. J Neurosci. 2001;21(16):6214–6220. doi:10.1523/JNEUROSCI.21-16-06214.2001 11487644 PMC6763179

[14] Bross M, Hackett M, Bernitsas E. Approved and Emerging Disease Modifying Therapies on Neurodegeneration in Multiple Sclerosis. IJMS. 2020;21(12):4312. doi:10.3390/ijms21124312 32560364 PMC7348940

[15] Lucchinetti CF, Popescu BFG, Bunyan RF, Inflammatory Cortical Demyelination in Early Multiple Sclerosis. N Engl J Med. 2011;365(23):2188–2197. doi:10.1056/NEJMoa1100648 22150037 PMC3282172

[16] Lassmann H. New concepts on progressive multiple sclerosis. Curr Neurol Neurosci Rep. 2007;7(3):239–244. doi:10.1007/s11910-007-0036-0 17488590

[17] Lycke JN, Karlsson JE, Andersen O, Rosengren LE. Neurofilament protein in cerebrospinal fluid: a potential marker of activity in multiple sclerosis. Journal of Neurology, Neurosurgery & Psychiatry. 1998;64(3):402–404. doi:10.1136/jnnp.64.3.402 9527161 PMC2170011

[18] Senol AD, Pinto G, Beau M, Alterations of the axon initial segment in multiple sclerosis grey matter. Brain Communications. 2022;4(6):fcac284. doi:10.1093/braincomms/fcac284 36451656 PMC9700164

[19] Duvick L, Southern WM, Benzow KA, Mapping SCA1 regional vulnerabilities reveals neural and skeletal muscle contributions to disease. JCI Insight. 2024;9(9):e176057. doi:10.1172/jci.insight.176057 38512434 PMC11141930

[20] Matilla A, Roberson ED, Banfi S, Mice Lacking Ataxin-1 Display Learning Deficits and Decreased Hippocampal Paired-Pulse Facilitation. J Neurosci. 1998;18(14):5508–5516. doi:10.1523/JNEUROSCI.18-14-05508.1998 9651231 PMC6793485

[21] Stone S, Wu S, Jamison S, Durose W, Pallais JP, Lin W. Activating transcription factor 6α deficiency exacerbates oligodendrocyte death and myelin damage in immune-mediated demyelinating diseases. Glia. 2018;66(7):1331–1345. doi:10.1002/glia.23307 29436030 PMC6019578

[22] Lei Z, Yue Y, Stone S, Wu S, Lin W. NF-κB Activation Accounts for the Cytoprotective Effects of PERK Activation on Oligodendrocytes during EAE. J Neurosci. 2020;40(33):6444–6456. doi:10.1523/JNEUROSCI.1156-20.2020 32661025 PMC7424865

[23] Zoghbi HY, Orr HT. Pathogenic Mechanisms of a Polyglutamine-mediated Neurodegenerative Disease, Spinocerebellar Ataxia Type 1. Journal of Biological Chemistry. 2009;284(12):7425–7429. doi:10.1074/jbc.R800041200 18957430 PMC2658037

[24] Liu H, Jin H, Yue X, PET Imaging Study of S1PR1 Expression in a Rat Model of Multiple Sclerosis. Mol Imaging Biol. 2016;18(5):724–732. doi:10.1007/s11307-016-0944-y 26975859 PMC5297893

[25] Hussien Y, Cavener DR, Popko B. Genetic inactivation of PERK signaling in mouse oligodendrocytes: Normal developmental myelination with increased susceptibility to inflammatory demyelination: Oligodendrocyte-Specific PERK Inactivation. Glia. 2014;62(5):680–691. doi:10.1002/glia.22634 24481666 PMC6342275

[26] Hou B, Yin J, Liu S, Inhibiting the NLRP3 Inflammasome with MCC950 Alleviates Neurological Impairment in the Brain of EAE Mice. Mol Neurobiol. 2024;61(3):1318–1330. doi:10.1007/s12035-023-03618-y 37702910 PMC10896958

[27] Shindler KS, Ventura E, Dutt M, Rostami A. Inflammatory demyelination induces axonal injury and retinal ganglion cell apoptosis in experimental optic neuritis. Experimental Eye Research. 2008;87(3):208–213. doi:10.1016/j.exer.2008.05.017 18653182 PMC2564281

[28] Craner MJ. Co-localization of sodium channel Nav1.6 and the sodium-calcium exchanger at sites of axonal injury in the spinal cord in EAE. Brain. 2004;127(2):294–303. doi:10.1093/brain/awh032 14662515

[29] Lo AC, Saab CY, Black JA, Waxman SG. Phenytoin Protects Spinal Cord Axons and Preserves Axonal Conduction and Neurological Function in a Model of Neuroinflammation In Vivo. Journal of Neurophysiology. 2003;90(5):3566–3571. doi:10.1152/jn.00434.2003 12904334

[30] Brambilla R, Morton PD, Ashbaugh JJ, Karmally S, Lambertsen KL, Bethea JR. Astrocytes play a key role in EAE pathophysiology by orchestrating in the CNS the inflammatory response of resident and peripheral immune cells and by suppressing remyelination. Glia. 2014;62(3):452–467. doi:10.1002/glia.22616 24357067

[31] Giraud SN, Caron CM, Pham-Dinh D, Kitabgi P, Nicot AB. Estradiol inhibits ongoing autoimmune neuroinflammation and NFκB-dependent CCL2 expression in reactive astrocytes. Proc Natl Acad Sci USA. 2010;107(18):8416–8421. doi:10.1073/pnas.0910627107 20404154 PMC2889572

[32] Wang X, Haroon F, Karray S, Deckert Martina, Schlüter D. Astrocytic F as ligand expression is required to induce T -cell apoptosis and recovery from experimental autoimmune encephalomyelitis. Eur J Immunol. 2013;43(1):115–124. doi:10.1002/eji.201242679 23011975

[33] Hao W, Decker Y, Schnöder L, Deficiency of IκB Kinase β in Myeloid Cells Reduces Severity of Experimental Autoimmune Encephalomyelitis. The American Journal of Pathology. 2016;186(5):1245–1257. doi:10.1016/j.ajpath.2016.01.004 26968344

[34] Sonar SA, Lal G. The iNOS Activity During an Immune Response Controls the CNS Pathology in Experimental Autoimmune Encephalomyelitis. Front Immunol. 2019;10:710. doi:10.3389/fimmu.2019.00710 31019516 PMC6458273

[35] Warnecke A, Musunuri S, N’diaye M, Nitration of MOG diminishes its encephalitogenicity depending on MHC haplotype. Journal of Neuroimmunology. 2017;303:1–12. doi:10.1016/j.jneuroim.2016.11.008 28011088

[36] Kim E, Park S, Choi N, Deficiency of Capicua disrupts bile acid homeostasis. Sci Rep. 2015;5(1):8272. doi:10.1038/srep08272 25653040 PMC4317698

[37] Jahan-Abad AJ, Karima S, Shateri S, Serum pro-inflammatory and anti-inflammatory cytokines and the pathogenesis of experimental autoimmune encephalomyelitis. Neuropathology. 2020;40(1):84–92. doi:10.1111/neup.12612 31709666

[38] Kemp K, Gordon D, Wraith DC, Fusion between human mesenchymal stem cells and rodent cerebellar Purkinje cells. Neuropathology and Applied Neurobiology. 2011;37(2):166–178. doi:10.1111/j.1365-2990.2010.01122.x 20819172 PMC4150530

[39] Valentin-Torres A, Savarin C, Hinton DR, Phares TW, Bergmann CC, Stohlman SA. Sustained TNF production by central nervous system infiltrating macrophages promotes progressive autoimmune encephalomyelitis. J Neuroinflammation. 2016;13(1):46. doi:10.1186/s12974-016-0513-y 26906225 PMC4763407

[40] Kemp K, Gray E, Mallam E, Scolding N, Wilkins A. Inflammatory Cytokine Induced Regulation of Superoxide Dismutase 3 Expression by Human Mesenchymal Stem Cells. Stem Cell Rev and Rep. 2010;6(4):548–559. doi:10.1007/s12015-010-9178-6 20683679

[41] Hou Y, Ryu CH, Park KY, Kim SM, Jeong CH, Jeun SS. Effective combination of human bone marrow mesenchymal stem cells and minocycline in experimental autoimmune encephalomyelitis mice. Stem Cell Res Ther. 2013;4(4):77. doi:10.1186/scrt228 23826999 PMC3854709

[42] Hidaka Y, Inaba Y, Matsuda K, Cytokine production profiles in chronic relapsing–remitting experimental autoimmune encephalomyelitis: IFN-γ and TNF-α are important participants in the first attack but not in the relapse. Journal of the Neurological Sciences. 2014;340(1–2):117–122. doi:10.1016/j.jns.2014.02.039 24655735

[43] Rosa JG, Hamel K, Sheeler C, Spatial and Temporal Diversity of Astrocyte Phenotypes in Spinocerebellar Ataxia Type 1 Mice. Cells. 2022;11(20):3323. doi:10.3390/cells11203323 36291186 PMC9599982

[44] Jukkola P, Guerrero T, Gray V, Gu C. Astrocytes differentially respond to inflammatory autoimmune insults and imbalances of neural activity. acta neuropathol commun. 2013;1(1):70. doi:10.1186/2051-5960-1-70 24252623 PMC3893391

[45] Remlinger J, Bagnoud M, Meli I, Modelling MOG antibody-associated disorder and neuromyelitis optica spectrum disorder in animal models: Spinal cord manifestations. Multiple Sclerosis and Related Disorders. 2023;78:104892. doi:10.1016/j.msard.2023.104892 37499337 PMC11792092

[46] Voskuhl RR, Peterson RS, Song B, Reactive Astrocytes Form Scar-Like Perivascular Barriers to Leukocytes during Adaptive Immune Inflammation of the CNS. J Neurosci. 2009;29(37):11511–11522. doi:10.1523/JNEUROSCI.1514-09.2009 19759299 PMC2768309

[47] Yadav SK, Ito N, Soin D, Ito K, Dhib-Jalbut S. Dimethyl Fumarate Suppresses Demyelination and Axonal Loss through Reduction in Pro-Inflammatory Macrophage-Induced Reactive Astrocytes and Complement C3 Deposition. JCM. 2021;10(4):857. doi:10.3390/jcm10040857 33669652 PMC7922578

[48] Mayo L, Trauger SA, Blain M, Regulation of astrocyte activation by glycolipids drives chronic CNS inflammation. Nat Med. 2014;20(10):1147–1156. doi:10.1038/nm.3681 25216636 PMC4255949

[49] Qu W, Johnson A, Kim JH, Lukowicz A, Svedberg D, Cvetanovic M. Inhibition of colony-stimulating factor 1 receptor early in disease ameliorates motor deficits in SCA1 mice. J Neuroinflammation. 2017;14(1):107. doi:10.1186/s12974-017-0880-z 28545543 PMC5445366

